# Generation of neutralizing and non-neutralizing monoclonal antibodies against H7N9 influenza virus

**DOI:** 10.1080/22221751.2020.1742076

**Published:** 2020-03-20

**Authors:** Fan Yang, Yixin Xiao, Rufeng Lu, Bin Chen, Fumin Liu, Liyan Wang, Hangping Yao, Nanping Wu, Haibo Wu

**Affiliations:** a State Key Laboratory for Diagnosis and Treatment of Infectious Diseases, and National Clinical Research Center for Infectious Diseases, the First Affiliated Hospital, School of Medicine, Zhejiang University, Hangzhou, People’s Republic of China; b Department of Emergency, the First Affiliated Hospital of Zhejiang Chinese Medical University, Hangzhou, People’s Republic of China

**Keywords:** Influenza virus, H7N9, monoclonal antibody, neutralization, antibody-dependent cellular cytotoxicity (ADCC)

## Abstract

The H7N9 viruses have been circulating for six years. The insertion of a polybasic cleavage site in the haemagglutinin (HA) protein of H7N9 has resulted in the emergence of a highly pathogenic (HP) avian influenza virus. Currently, there are limited studies on neutralizing monoclonal antibodies(mAbs) against HP H7N9 AIVs. In this study, mice were immunized with inactivated H7N9 vaccine of A/ZJU01/PR8/2013 to produce murine mAbs. Finally, two murine mAbs against the HA of low pathogenic (LP) virus were produced and characterized. Characterization included determining mAbs binding breadth and affinity, in vitro neutralization capacity, and potential in vivo protection. Two of these mAbs, 1H10 and 2D1, have been identified to have therapeutic and prophylactic efficacy against the HP strain in mouse passive transfer-viral challenge experiments. The mAb 1H10 was most efficacious, even if the treatment-time was as late as 72 h post-infection, or the therapeutic dose was as low as 1 mg/kg; and it was confirmed to have haemagglutination inhibition and neutralizing activity on both LP-and HP-H7N9 strains. Further study indicated that the protection provided by 2D1 was mediated by antibody-dependent cellular cytotoxicity. The mAbs described here provide promising results and merit further development into potential antiviral therapeutics for H7N9 infection.

## Introduction

Avian influenza virus (AIV) is an enveloped, segmented, negative-strand RNA virus of the Orthomyxoviridae family [1]. The primary host of AIV are birds, but occasionally the virus can breach the species barrier from domestic poultry to humans, such as H5N1, H6N1, H7N3, H7N9, H9N2 and H10N8 [2–5]. Among these strains, H5N1 and H7N9 infections have received extensive attention due to their alarmingly high mortality rate [6].

The H7N9 virus was first isolated from patients in Eastern China in early 2013 and has been reported to cause five epidemic waves in China [7]. The general clinical manifestations of H7N9 patients are flu-like symptoms, but it is prone to cause acute respiratory distress syndrome or other complications especially in the elderly and children [8]. Up to now, H7N9 has been confirmed cause more than 1500 individual infections with a mortality rate of approximately 40% [9]. In addition, the circulating H7N9 viruses have evolved in these successive waves through mutation accumulation and genomic reassortment [10]. Highly pathogenic (HP) H7N9 viruses were identified in the fifth wave in 2017, and have caused fatal outcomes in China [11]. Control of these viruses remains an important point of concern for global human health.

Currently, vaccination remains the most effective measures to reduce morbidity and mortality caused by influenza virus infection [12]. However, there is no commercially available vaccine for H7N9 infection in humans and the primary therapeutic treatments remain supportive medical care and neuraminidase inhibitors (NAIs). Previous studies have shown, NAIs are limited by their short treatment time-window (within 48h after symptom onset) and the emergence of drug-resistant virus [13]. In March 2018, baloxavir marboxil, a new anti-influenza drug, was firstly introduced in Japan with the treatment of uncomplicated acute influenza patients within 48 h [14]. Similar to NAIs, resistant mutation (PA-I38T/M/F) to baloxavir marboxil frequently observed in patients (1.1–19.5%) within 3–5 days of treatment [15, 16]. Therefore, it is important to establish an alternative antiviral method. Therapeutic monoclonal antibodies (mAbs) have become a prospective part of infectious diseases due to their specificity, limited off-target effects, and favourable safety profile. Primary examples of mAbs as treatment are palivizumab against respiratory syncytial virus and ZMapp against Ebola virus [17, 18].

AIV membranes contain two major surface proteins, haemagglutinin (HA) and NA, and the majority of the neutralizing antibodies produced by vaccination or viral infection are targeted against the HA protein [19]. The mature HA protein is comprised of two disulfide-linked subunits, HA1 and HA2. The HA1 subunit constitutes the HA head domain and contains the sialic acid receptor binding site (RBS), while the HA2 subunit and a portion of HA1 form a stalk structure. The HA globular head domain mediates receptor binding and the HA stalk domain mediates host-virus membrane fusion, both of which are critical in its life cycle [20]. Antibodies target the globular HA head or a conserved site in the HA stem to anti AIVs. The anti-head antibodies are in general strain or clade specific, due to the highly variable nature of its targeted residues. The anti-stem antibodies had been shown to be extremely broad through antibody-dependent cellular cytotoxicity studies (ADCC) [21].

Here, two murine mAbs against the HA of the H7N9 virus were produced and characterized, with cross-neutralizing activity against LP and HP of H7N9 viruses in vitro. Further studies showed the mAbs elicted protective effects in mice from lethal challenge when administered in either a prophylactic or therapeutic setting, which provide evidence that these mAbs could be a suitable prophylactic and/or therapeutic candidates against a potential pandemic.

## Materials and methods

### Cells, viruses and vaccines

Madin-Darby canine kidney (MDCK) cells line was obtained from the ATCC (Rockville, MD, USA) and passaged in Dulbecco’s Modified Eagles Medium (complete DMEM, Gibco) which was supplemented with 10% foetal bovine serum (FBS, Gibco) and antibiotics solution consisting of 10,000 units per ml of penicillin and 10,000 µg/ml of streptomycin (Pen Strep, Gibco).

Wild-type influenza virus strains, A/duck/Zhejiang/DK10/2013(H7N3), A/chicken/Jiangxi/C25/2014(H7N7), A/Zhejiang/DTID-ZJU01/2013(ZJU01, H7N9) and A/Guangdong/HP001/2017(HP001,H7N9) were isolated from poultries or patients from 2013 to 2018 [22–25], and A/California/7/2009(H1N1) and A/Texas/50/2012(H3N2) were stored at our labs. All viruses were propagated in 11-d embryonated chicken eggs, and virus titres were determined by a standard tissue culture infectious dose 50 (TCID50) assay as described previously [26]. All research with H7N9 were conducted under BSL3 laboratory containment conditions.

The H7N9 vaccine was inactivated H7N9 avian influenza vaccine (split virion) containing 30 µg/ml HA of A/ZJU01/PR8/2013 and provided by the Zhejiang Tianyuan Bio-Pharmaceutical Co., Ltd. [25]. And the A/ZJU01/PR8/2013 was reassortant H7N9 strains which harboured the HA and NA genes of A/Zhejiang/DTID-ZJU01/2013 and the six internal protein genes of A/PR/8/34 [27].

### Production and characterization of murine mAbs

MAbs were produced as described previously [28]. Briefly, 6-week-old, female, BALB/c mice (Shanghai Laboratory Animal Center) were immunized with two intramuscular injection of H7N9 virus vaccine in Quick Antibody adjuvant (Biodragon, China) with 2-week interval. The mice then received an additional intravenous injection of the same viral antigen 3 days without adjuvant before the fusion of splenocytes with SP2/0 myeloma cells [29]. After cell fusion, hybridoma culture supernatants were screened by enzyme-linked immunosorbent assay (ELISA) [30]. Through limiting dilution, positive hybridoma lines were cloned, expanded and further sub-cultured. Hybridoma cell clones were expanded significantly, harvested, and injected intraperitoneally in pristane-primed BALB/c mice [29]. The ascites was collected and purified by protein-G column (GE) according to the manufacturer’s instructions. The concentrations of purified mAbs were determined by Nanodrop 2000 (Thermo), and were stored at −80°C. The subclass and type of mAbs were determined by using the Mouse monoclonal antibody isotyping kit (Bio-Rad) as directed by the manufacturer’s instructions. The heavy and light chains of the hybridomas are sequenced by Genscript (Nanjing, China).

### Direct ELISA

Purified proteins were used to coat 96-well plates (30 ng/well) overnight at 4°C. Culture supernatants from individual hybridomas were added to each well (100 µl/well) followed by horseradish peroxidase (HRP)-conjugated goat anti-mouse IgG (Novus). The values of the optical density (OD) of substrate reactions were read at 450 nm on a plate reader (Bio-Rad).

The binding ability of mAb was measured by ELISA as described previously [31,32]. MAbs were serially two-fold diluted at an initial concentration of 10 µg/ml and detected by ELISA as described above.

### Immunofluorescence assay (IFA)

The IFA was performed as described previously [29]. Monolayers of MDCK cells were infected at a multiplicity of infection (MOI) of 0.5 with virus. At 24 h post-infection, the cells were fixed with 4% paraformaldehyde and permeabilized with 0.5% Triton X-100. After washing with phosphate-buffered saline (PBS), cells were blocked with 3% bovine serum albumin (BSA) solution. The blocking solution was removed and 10 µg/ml of each mAb was incubated for 1 h at room temperature. Fixed cells were rinsed, and then incubated with a 1:1000 dilution of the goat anti-mouse IgG heavy plus light chain (H + L)–Alexa Fluor 488 (Abcam). Cells were rinsed again, and antibody binding was evaluated using an Image-Pro Plus image system with a GFP imaging cube. Representative images were taken and appropriately labeled by experiment. Isotype antibodies (IgG1 or IgG2a) served as negative controls for the IFA experiments.

### Haemagglutination inhibition (HI) assay

The viruses were titrated using HA of 1% chicken red blood cells (RBCs) before the HI assay was performed [33]. Control negative and positive sera were treated with receptor destroying enzyme (RDE) and inactivated at 56°C for 30 min [28]. The mAbs and inactivated sera were 2-fold serially diluted in PBS in a 96-well V-bottom plate and were mixed with 4 HA units (HAU) of virus per well. After 30 min room temperature incubation, HI was titrated with 1% chicken RBCs [34].

### Virus microneutralization (MN) assay

The MN assay was performed as previously described [35]. MAbs were serially two-fold diluted from 10 to 0.015 µg/ml, mixed with an equal volume of 100 TCID50 of virus and incubated for 2 h at 37°C. The virus-antibody mixture was then added to confluent MDCK monolayers in 96-well plates and supplemented with 2 µg/ml TPCK-trypsin (Worthington) and incubated for 72 h at 37°C. Each sample consisted of four replicates, and the mAbs titres required to reduce virus replication by 50% were determined by an HA assay [33], following the Reed and Muench method [26].

### Evaluation of mAbs for its prophylactic and therapeutic protective activities in mice

The animal studies were performed in accordance with the recommendations of the Office International des Epizooties [36] and approved by the First Affiliated Hospital, School of Medicine, Zhejiang University (No. 2017–2015). Seven- to eight-week-old BALB/c mice were infected intranasally with various doses of HP001 to determine the 50% mouse lethal dose (MLD50) [37]. Mice were weighed on the day of virus challenge and monitored daily for 14 days for weight loss and survival (mice with a body weight loss of ≥25% were euthanized). Weight loss and survival data were analysed using Prism 5 software.

To determine the prophylactic efficacy of the mAbs, mice in groups of 14 were administered either mAbs intraperitoneally at single doses of 0.3, 1, 3, 10 and 30 mg/kg or a mouse isotype control IgG (Solarbio) at 30 mg/kg in 200-µl volume. After 6 h-injection, mice were inoculated intranasally with 3 × MLD50 of HP001 in a 50-µl volume. All mice were weighed and monitored daily [38]. For the lung titre study, three mice from each group were euthanized at 3 d and 6 d post-infection respectively. Half of the lungs were kept in 10 ml formalin for histopathological analysis, and the other half were homogenized in PBS. Virus titres in lung homogenates were determined using the TCID50 assay.

For the study of therapeutic efficacy, mice were infected intranasally with 5 × MLD50 of HP001 in 50-µl volumes. At 6 h (D0), 24 h (D1), 48 h (D2) or 72 h (D3) post-infection, groups of 5 mice were injected intraperitoneally with 1, 3 and 10 mg/kg of mAbs. Mice in the control group were administered isotype control IgG of 10 mg/kg at 6 h post-infection. Mice were observed and weighed daily.

### Generation of antibody escape mutants

The conformational epitopes recognized by mAbs were mapped by characterization of escape mutants as described previously [39]. Briefly, 100 TCID50 of virus was mixed with 10 µg/ml of mAb, isotype control mAb or PBS for 1 h at room temperature. Then mixtures were inoculated into 11-day-old embryonated chicken eggs. The allantoic fluid was harvested and tested using an HA assay [33]. The positive allantoic fluid was again mixed with mAbs and passaged in eggs as described previously. The selection process was repeated three times with increasing mAbs concentrations (10, 20 and 40 µg/ml) in the medium. Allantoic fluid was harvested and RNA was extracted using Trizol LS reagent (Life Technologies) as described previously [23]. The HA gene and NA gene segments were amplified by reverse transcription-PCR (RT–PCR) with segment–specific primers as described previously [40] and sequenced to determine the amino acid changes.

### Generation of recombinant viruses

Plasmid-based reverse genetics was performed as described previously to generate the recombinant H7 viruses expressing HAs carrying those mutations [26, 41]. Briefly, the cDNA of the mutant virus was synthesized, amplified and cloned into pHW2000 plasmid [41]. Single point mutations were inserted as described previously [42]. Six plasmids of A/Puerto Rico/8/1934(H1N1) (PR8) and mutant plasmid were cotransfected to 293T cells using Lipofectamine 2000 Reagent (Invitrogen, United States) and incubated for 48 h at 37°C. The culture supernatant was harvested and then propagated in embryonated eggs [43].

### Flow cytometry of infected target cells

MDCK cells infected by H7N9 virus at an MOI of 0.5 for 16 h were used as target cells [35]. Purified mAbs or isotype control antibody were incubated with the target cells at a concentration of 5 µg/ml at 4°C for 1h. After washing three times with fluorescence-activated cell sorting (FACS) buffer (1% BSA in PBS), the cells were then incubated with goat anti-mouse IgG–Alexa Fluor 488 (Abcam) at 4°C for 1 h. Followed by washing twice, the stained cells were analysed on a flow cytometer (BD), and the results were analysed using the FlowJo software (BD).

### ADCC assay

The ADCC activity of mAbs was determined using a flow cytometry-based assay as described previously [44], with minor modifications. Briefly, target cells were prepared and harvested as described above. To label the target cells with Carboxyfluorescein succinimidyl ester (CFSE, BD) was used according to the manufacturer’s instructions. The target cell suspension (5 × 10^5^ cells/ml) was mixed with an equal volume of serially diluted mAb (40, 10 and 2.5 µg/ml). After incubation, mouse NK cells were added to the target cell-mAb mixture. Following 6h of incubation, dead cells were stained by 7-AAD (eBioscience) according to the manufacturer’s instructions. Two control groups were additionally set up, including target cells alone (spontaneous lysis) and target cells with Triton X-100 added (maximum fluorescence). A total of 10,000 target cells were analysed on a BD flow cytometer, and the relative proportions of four identifiable cell populations determined via FlowJo software analysis. The percentage ADCC activity of tested mAbs was expressed as described previously [35].

## Results

### Murine mAbs bind to the HA of H7N9 virus

**Figure 1.**
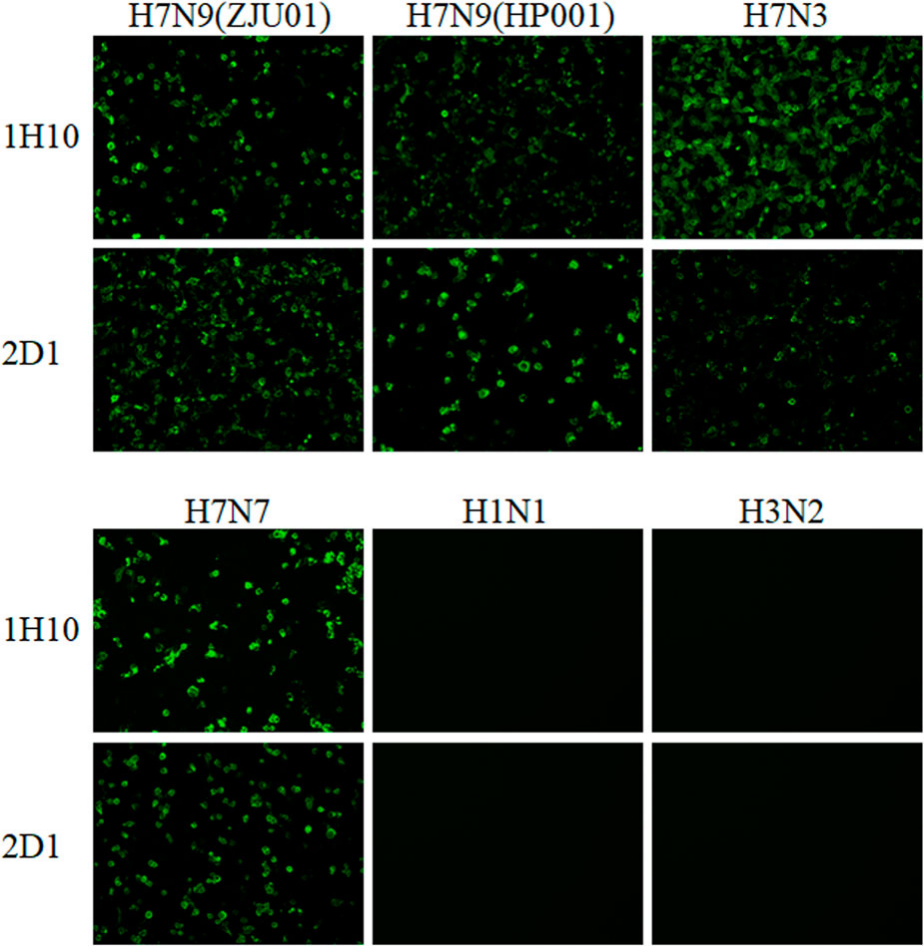
Immunofluorescence assay to measure the binding of mAbs to AIVs. MDCK cells infected with H7N3, H7N7, ZJU01, HP001, H1N1 and H3N2 viruses were incubated with mAbs. Binding by mAbs was detected by Alexa Fluor 488 (green) conjugated secondary antibodies. The binding of mAbs to viruses was detected by immunofluorescence.

**Figure 2.**
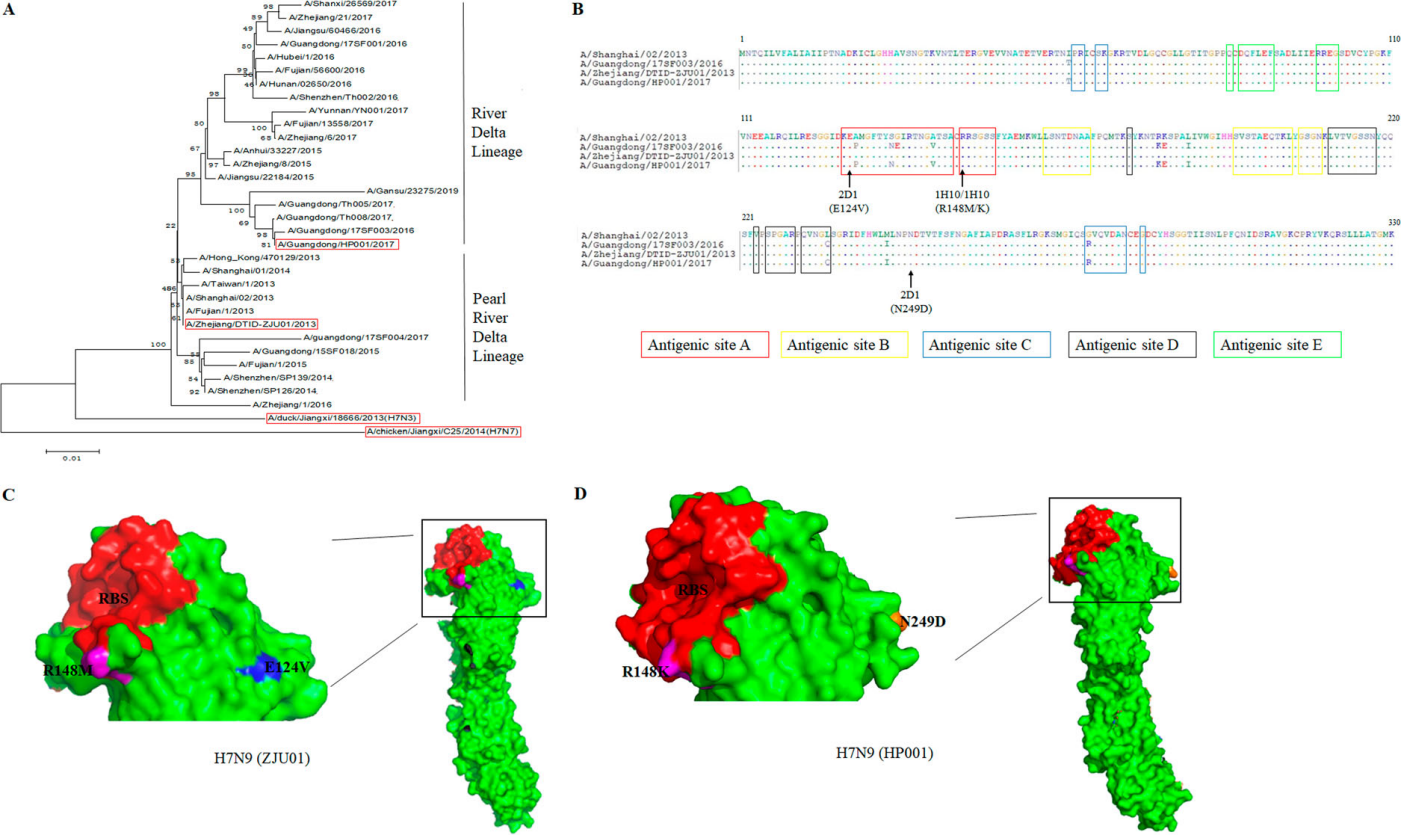
Phylogenetic analysis and epitope mapping. (A) A phylogenetic analysis of the HA genes of representative H7N9 viruses collected from 2013 to 2019 in the GISAID. The evolutionary tree was inferred using the neighbor-joining method and was generated in MEGA6. The viruses used in this study are indicated in the red box. (B) The sequence alignments of the HA gene of H7N9 virus and escape mutations of mAbs (1H10 and 2D1). The “.” symbol indicates sequence identity with the A/Shanghai/02/2013 strain and antigenic sites A, B, C, D and E were labeled. Escape mutations selected by mAbs in ZJU01 are indicated in black and by an arrow, while escape mutations selected by mAbs in HP001 are indicated in red and by an arrow. (C) A graphical representation of the crystal structure of A/shanghai/2/2013 (PDB: 4LN6), and the RBS was indicated in red. MAb 1H10 (purple) and 2D1 (orange) escape mutation regions against ZJU01 are represented by the corresponding colour. (D) A graphical representation of the crystal structure of A/Guangdong/17SF003 /2016 (PDB: 6D7U), and the RBS is highlighted in red. MAb 1H10 (purple) and 2D1 (orange) escape mutation regions against HP001 are represented by the indicated colour.

Two murine mAbs (1H10 and 2D1) were produced in our study using hybridoma technology [28]. The isotypes of the two mAbs were detected and listed in Table 1. The mAb 1H10 was IgG1 isotype and mAb 2D1 was IgG2a. These two mAbs have strong binding to HA of ZJU01 (Table 1). The heavy and light chains of the 1H10 and 2D1 were showed in the Table 1. The binding specificity of mAbs was verified by IFA (Figure 1). The IFA was performed to assess whether the mAbs can bind to H7N9 in its native confirmation as it is expressed on the surface and inside of infected cells. The IFA results revealed that the mAbs have binding specificity for the H7 subtype virus. Phylogenetic analysis of the HA gene of H7N9 viruses (Figure 2(A)) indicated that the ZJU01 and HP001 were representative strains from the lineages of the Yangtze River Delta and the Pearl River Delta, respectively. And the sequence of HP001 was highly similar (>99.9%) with the candidate vaccine strain A/Guangdong/17SF003/2016 as determined by sequence alignment (Figure 2(A and B)).

**Table 1.** The isotype and affinity of two mAbs against H7N9 virus.

Names	Isotype^a^		ELISA^b^ (µg/ml)	Heavy chain		Light chain	
	Subclass	Type		V-GENE and allele	CDR3^c^	V-GENE and allele	CDR3
1H10	IgG1	κ	1×10^−3^	IGHV1-39*01 F	NFDFDY	IGKV1-133*01 F	VQGTHFPYT
2D1	IgG2a	κ	2×10^−4^	IGHV14-3*02 F	AFNWDRFTY	IGKV10-96*01 F	QQANTLPYT

^a^The isotypes of mAbs were detected by mouse monoclonal antibody isotyping kit according to the manufacturer’s instructions

^b^The ELISA was performed using the HA protein of A/Zhejiang/DTID-ZJU01/2013.

^c^Complementarity-determining region.

### The neutralizing activities of mAbs against H7N9 virus in vitro

To further assess whether the mAbs can bind on or near the receptor binding site of the HA of H7N9 in a manner that competes with binding with the virus cell surface receptor, HI assays were performed using ZJU01 and HP001 as described previously [33,39]. The mAb 1H10 showed strong HI activity against ZJU01, H7N3 and H7N7 at low minimal HI concentrations. Meanwhile the 1H10 antibody exhibited HI activity against the HP001 and inhibited activity at a low minimal HI concentration. And mAb 2D1 had moderate HI activity against the H7N9 AIVs. In addition, 1H10 and 2D1 had weak HI activity against H3N2 AIVs, probably due to H3N2 and H7N9 AIVs are both group 2 viruses, and share some antibody binding epitopes in the HA domain [44]. MAb 1H10 raised against the ZJU01 virus can bind to the H7 HA of HP001 with similar minimal binding concentrations (Table 2).

**Table 2.** MAbs HI and neutralization activities against influenza viruses.

Names	HI activity^a^ (µg/ml)						Neutralizing IC50^b^ (µg/ml), mean ± SD			
	ZJU01	HP001	H1N1	H3N2	H7N3	H7N7	ZJU01	HP001	H7N3	H7N7
1H10	0.31	0.9	>5000	5000	0.31	0.62	0.15 ± 0.02	0.075 ± 0.07	>5000	>5000
2D1	50	62.5	>5000	5000	>5000	>5000	>5000	>5000	>5000	>5000

^a^HI titres are expressed as the lowest concentrations of purified mAbs that completely inhibited haemagglutination.

^b^IC50, half-maximal inhibitory concentration. Neutralization was detected using a haemagglutination assay.

The neutralizing activity in vitro was assessed using MN assays as described in Materials and Methods. The HI-active mAb 1H10 neutralized ZJU01 and HP001 and the IC50 was 0.15 µg/ml and 0.075 µg/ml, respectively. The mAbs 2D1 did not show neutralizing potential for the viruses at the tested concentrations.

### The protective effects of mAbs in vivo

To test if the mAbs have protective activity in vivo, we assessed the efficacy in prophylaxis and therapy of mAbs against HP001 virus in mice, and we found that 1H10 and 2D1 protect mice from lethal challenge (data not show).

**Figure 3.**
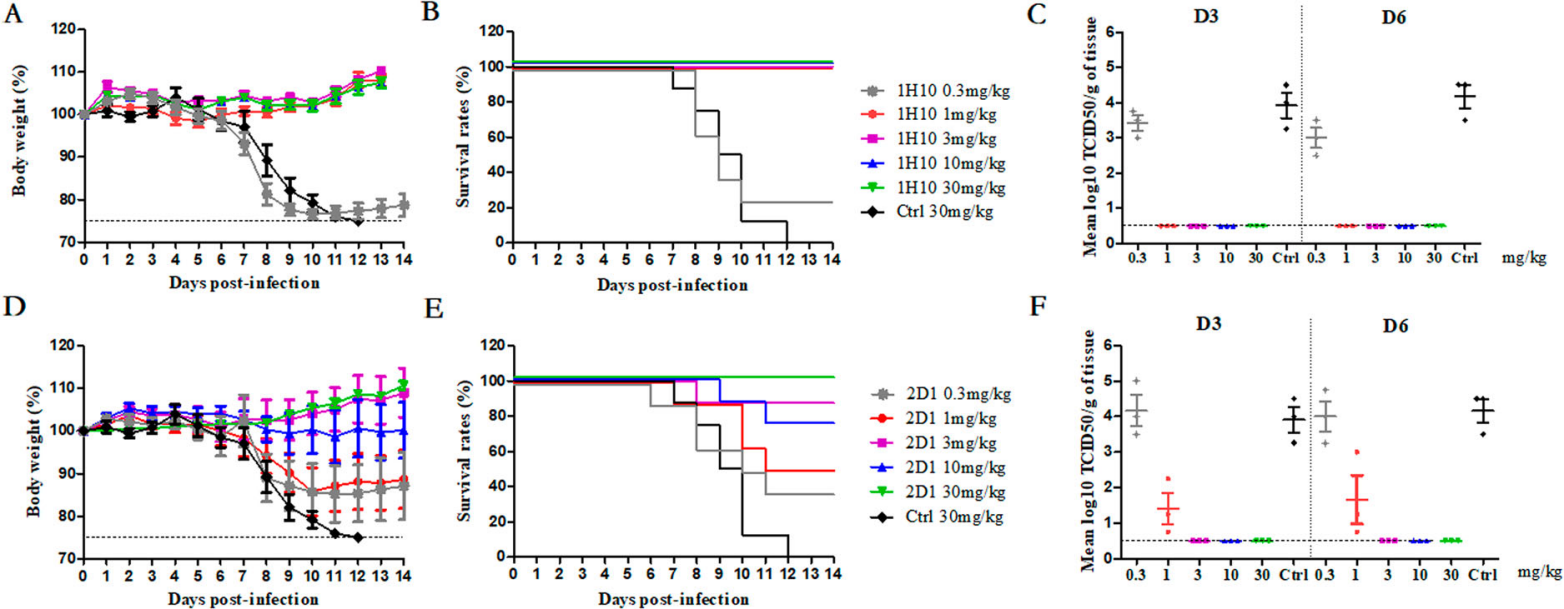
In vivo prophylactic efficacy of mAbs in mice. Prophylactic efficacy of mAbs 1H10 (A) and 2D1 (D) against lethal challenge with 3 × MLD50 of HP001. The survival curves of BALB/c mice (*n* = 8 per group) treated with 1H10 (B) and 2D1 (E) (0.3, 1, 3, 10, or 30 mg/kg) or isotype IgG (30 mg/kg) 6 h before lethal challenge. The virus titres in the lungs of the mice treated with mAbs 1H10 (C) and 2D1 (F) prophylactically or isotype IgG therapeutically were determined on days 3 and 6 post-infection.

To further evaluate the efficacy of mAbs against H7N9, including prophylaxis and therapy, 1H10 and 2D1 were evaluated in a mouse model of HP001. In the prophylaxis study (Figure 3), mAbs 1H10 and 2D1 conferred protection in a dose-dependent manner. MAb 1H10 provided 100% protection at a low dose of 1 mg/kg to 30 mg/kg, and the protective effect improved with increasing dose. In contrast, a higher dose of 30 mg/kg was required for 2D1 to be 100% protective. In addition, compared with 3 mg/kg, the 2D1 10 mg/kg results in less protection, more weight loss and more mortalities rate, which may be related to the individual differences among mice. No significant weight loss was observed in the 30 mg/kg dose and the survival rate was 100%, while the survival rate in the 0.3 mg/kg dose was only 40%. The viral loads in the lungs were determined, and 1H10-treated mice resulted in a minimum of a 3-fold reduction in viral titre compared to control mice, and virus titres in the lungs were undetectable when the dose is greater than 3 mg/kg (Figure 3). The viral titre in the lungs of 2D1-treated mice was reduced in varying degrees. Taken together, the mouse model studies indicated that 1H10 and 2D1 significantly reduce viral replication in the lungs of infected mice, and correlated with increased animal survival.

**Figure 4.**
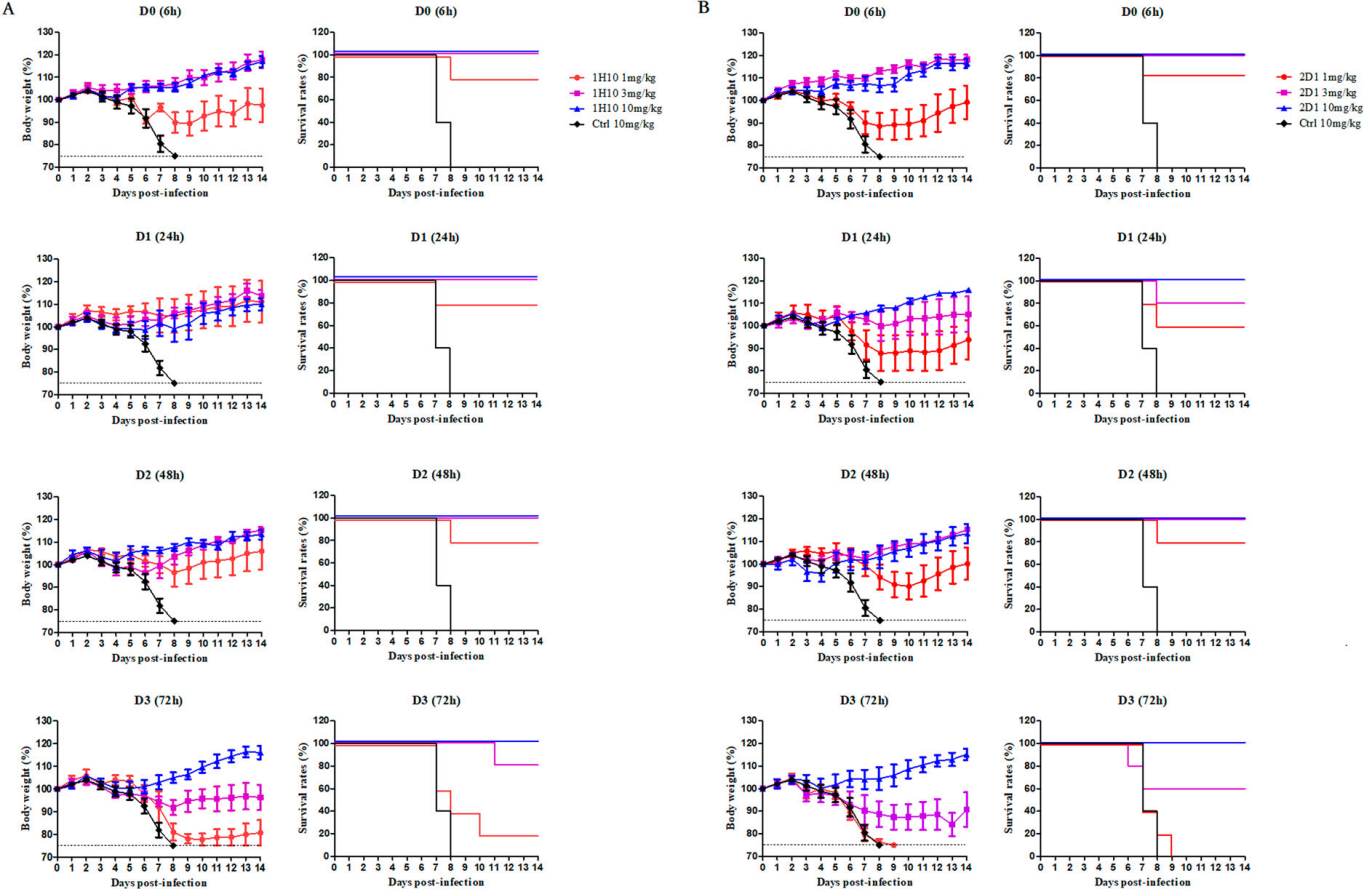
In vivo therapeutic efficacy of mAbs in mice. Prophylactic efficacy of mAbs 1H10 (A) and 2D1 (B) against lethal challenge with 5 × MLD50 of HP001. The weight loss and survival curves of BALB/c mice (*n* = 5 per group) treated with 1H10 and 2D1 (1, 3, or 10 mg/kg) or control IgG antibody (10 mg/kg) at 6, 24, 48 and 72 h after lethal challenge.

In the therapeutic experiments, 1H10 and 2D1 were able to protect mice against a lethal challenge of H7N9 virus; this protection varied with dose and treatment time (Figure 4). When H7N9-infected mice were treated with a single dose of 10 mg/kg, 1H10 and 2D1 provided 100% protection even at 72 h post-infection. When H7N9-infected mice were treated 6h post-infection, 1H10 and 2D1 showed 80% protection at a concentration of 1 mg/kg. When mAb dosage administration was delayed or dosage reduced, the protective effect of mAbs gradually weakened.

**Figure 5.**
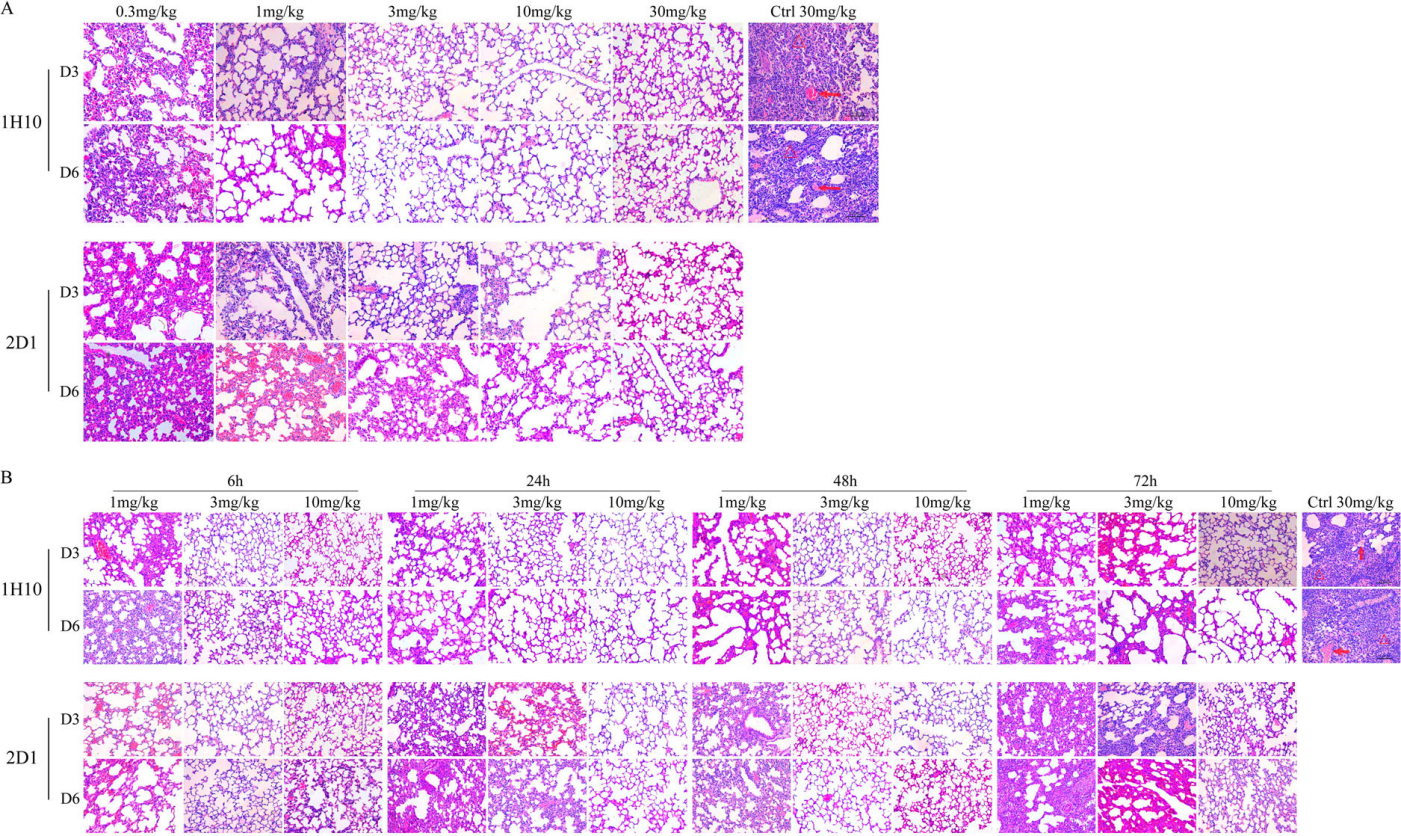
Histological analysis of lungs from H7N9-infected mice treated with antibodies. (A) The preventive experiment. (B) The therapeutic experiment. Significant infiltration of erythrocytes and inflammatory cells (triangle) and vascular congestion (arrow) could be observed in symptomatic lung tissues.

Histopathological analysis results of infected mice were consistent with therapeutic experiments (Figure 5). In both the preventive and therapeutic experiments, lung tissues of the infected mice treated with the control IgG had moderate multifocal interstitial inflammatory hyperaemia and exudative pathology changes at 3 d post-infection. At 6 d post-infection, lesions in the lung tissue became larger, with transmural infiltration of inflammatory cells and respiratory epithelial cell necrosis. In prophylactic experiments, 1H10 and 2D1 high dose treated mice had only mild pulmonary interstitial pneumonia and alveolitis. In therapeutic experiments, the pathological change of lung tissue in infected mice treated with mAbs low dose 72 h post-infection had similar virus induced lesions as the isotype control IgG-treated mice, and pathologic signs were weak in mice treated with high-dose mAbs 6 h post-infection. The collection of these results demostrate that the mAbs are protective in vivo, reduced lung virus titres, and could be applied as prophylactics and therapeutics.

### Epitope identification by selection of monoclonal antibody resistant mutants (MARMs)

After three virus passages under mAbs selection, mutant viruses that escaped from each mAb were obtained and analysed by direct sequencing of their HA genes (Table 3 and Figure 2(B)). No mutation sites were detected at HA in ZJU01 and HP001 which co-cultured with isotype control mAb or PBS. Previous studies have demonstrated that there are five conventional antigenic sites in H3N2 influenza virus HA protein (A, B, C, D and E), all of which have been extensively characterized [45]. An amino acid substitution was detected at HA residue 148 by mAb 1H10 in ZJU01 (R148M) and HP001 (R148K). MAb 2D1 treatment resulted in an E129V mutation in the ZJU01 and N249D in the HP001. The mutation R148K/M and E129V are both located in antigenic site A. As reported previously [46], antigenic site A is a common and conserved in all H7 HA sequences, including North American and Eurasian lineages. A total of 1391 HA genes of H7N9 from 2013 to 2019 have been obtained from the GISAID and were aligned using the software MEGA 6.0 [47]. Additionally, it was found that 565 (40.62%) HA proteins were mutated at position 148 in the amino acid sequence of the antigenic site A (RRSGSS), and most (39.91%) changed arginine (R) to lysine (K). This mutation occurred with significant abundance from 2016 to 2017 (96.40%), which may be related to the antigenic changes of H7N9. Meanwhile no mutations (E129V) in the HA gene were found at the position 129 in the antigenic site A.

**Table 3.** Conservation of H7N9 HA residues substituted in MARMs^a^

Names	Residue in MARMs	Residue(s) (%) observed in circulating H7N9 strains
1H10	ZJU01: R148M^b^	R (59.38%), K (39.91%), X (0.36%), G (0.14%), M (0.14%), N (0.07%)
	HP001: R148K^c^	
2D1	ZJU01: E124V	E (100%)
	HP001: N249D	N (100%)

^a^A total of 1391 H7N9 HA protein sequences from the GISAID from 2013 to 2019 were analysed.

^b^Escape mutations selected by mAbs against ZJU01 are indicated.

^c^Escape mutations selected by mAbs against HP001 are indicated.

**Figure 6.**
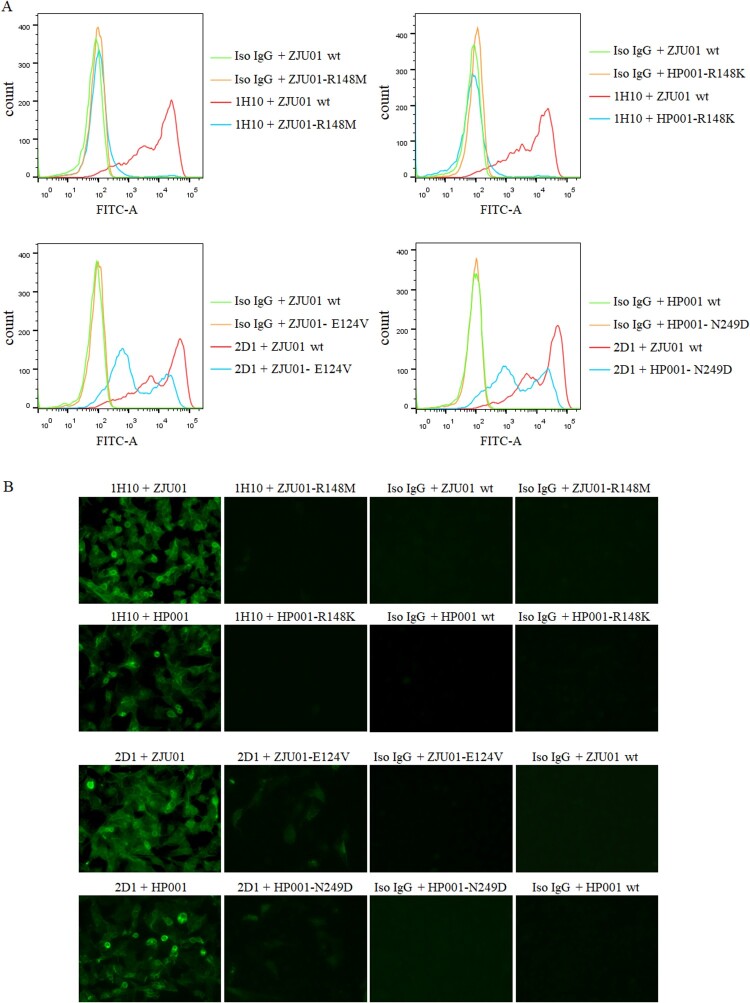
The binding of mAbs to mutants. (A) Binding of H7N9 HA mutants to mAbs were measured by flow assay. 1H10, 2D1 or control IgG antibody were tested at 5 µg/ml. (B) Immunofluorescence assay was performed to compare the binding of mAb to corresponding mutants. MDCK was infected (MOI 0.01) with wild type (WT) H7N9 or the mutants. Binding by mAbs was detected by Alexa Fluor 488 (green) conjugated secondary antibodies.

To further confirm the contribution of each of the above residues, four HA mutant viruses containing R148M (ZJU01-R148M), R148K (HP001-R148K), E124V (ZJU01- E124V) and N249D (HP001- N249D) were generated. MDCK cells were infected by these four HA mutant viruses, wild type ZJU01 and wild type HP001 and analysed by an IFA and flow assay. As Figure 6(A) showed, mAb 1H10 completely lost binding to ZJU01-R148M mutant and the HP001-R148K mutant, while ZJU01- E124V mutant and HP001- N249D mutant keep the partial binding with mAb 2D1. The result of flow assay was showed in Figure 6(B), no fluorescence signals were observed for the 1H10 against ZJU01-R148M mutants and the HP001-R148K mutants. In the other hand, compared with wt ZJU01 and wt HP001, low fluorescence signals were still observed in the 2D1 against ZJU01-E124V and HP001-N249D, but they are significantly weakened. These results suggested that the substitution of R148 in the HA protein with M or K in the escape virus completely prevent mAb 1H10 from binding to the H7N9 virus. In contrast, the substitution of E124V and N249D was found to have little effect on mAb 2D1.

### The ADCC effect of mAbs

**Figure 7.**
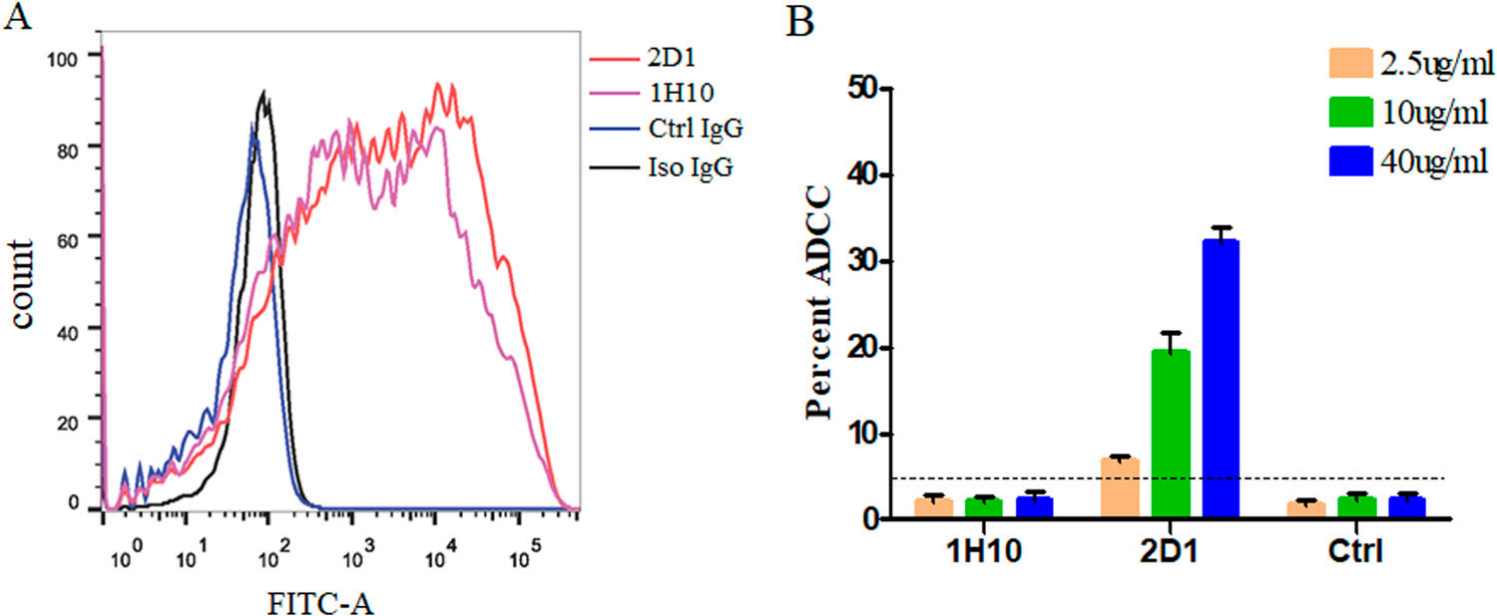
ADCC activity of mAbs against the H7N9 viruses. (A) Binding of mAbs to HP001-infected MDCK cells by flow cytometry. 1H10, 2D1 or control IgG antibody were tested at 5 µg/ml. (B) ADCC activities of 1H10, 2D1 or control IgG antibody against infected MDCK cells. Error bars represent the mean ± SEM. The indicated antibodies were tested at concentrations of 40, 10 and 2.5 µg/ml. The control IgG (1E7), the anti-syphilis antibody, was used as a control IgG. *P < 0.05, compared to the control IgG group.

The strong binding of 1H10 and 2D1 to H7N9-infected target cells was confirmed by flow cytometry (Figure 7(A)), which is consistent with the ELISA results. We found that 2D1 protected mice from lethal challenge on influenza even if it has no neutralization activity against HP001 in vitro. The ADCC activities were measured by a flow cytometry-based ADCC assay. An increase in the percent cytotoxicity against HP001 was observed in the presence of 2D1, while the 1H10 did not produce any ADCC activity (Figure 7(B)).

## Discussion

With the emergence of HP-H7N9 and drug-resistant strains in human infections, the H7N9 virus continues to be a serious threat to public health. MAbs play an important role in human infection disease treatment [48]. MAbs are a promising treatment potential and here we develop neutralizing antibodies for the prevention and control of the potentially fatal H7N9 infections in humans.

**Figure 8.**
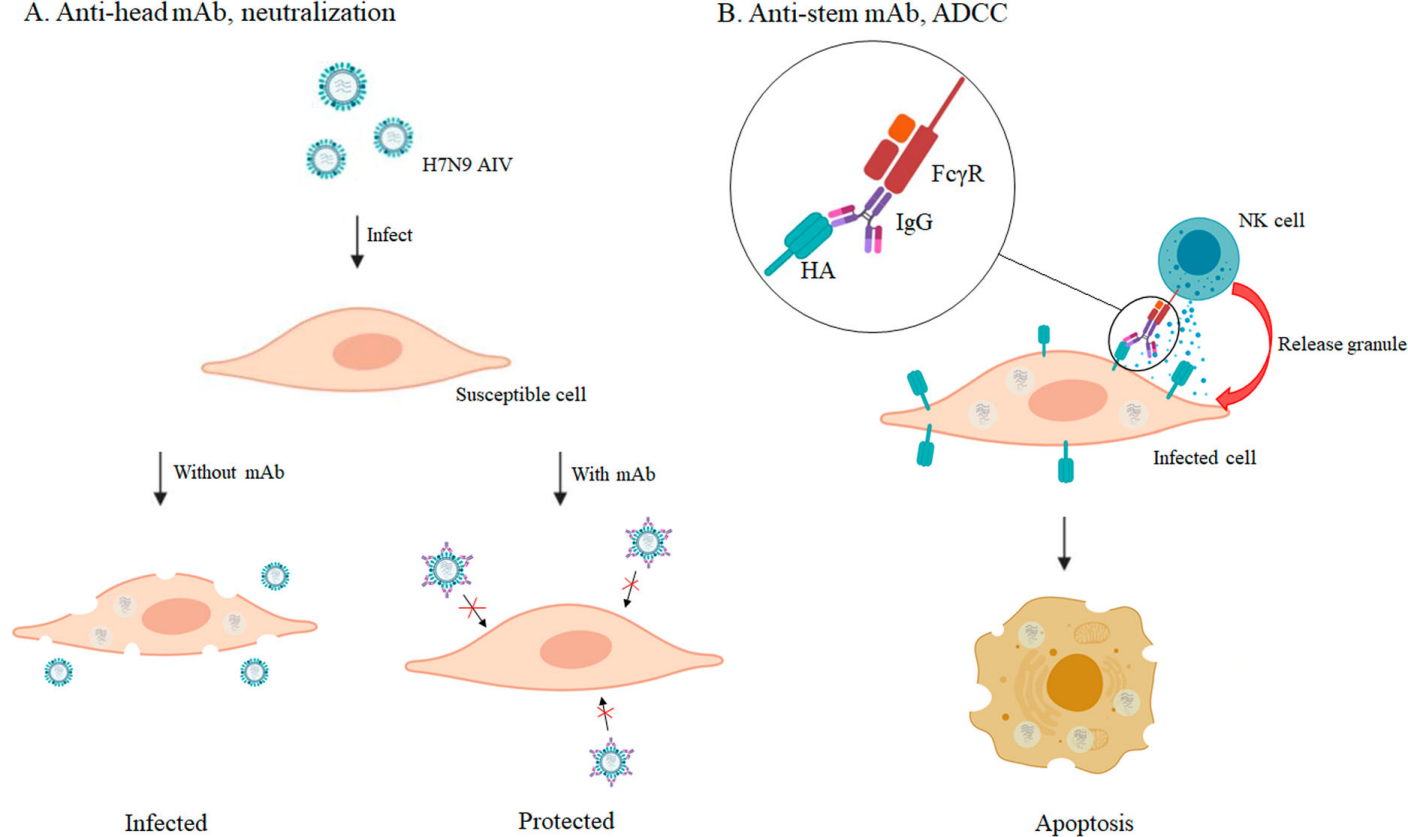
The protective mechanism of mAbs. (A) The anti-head mAb interacts with HA and interferes with the attachment of the virus to the susceptible cell. (B) The anti-stem mAb marks infected cells to attract natural killer (NK) cells and cellular destruction via the process of ADCC.

Here we describe, five murine mAbs specific to H7N9 produced by hybridoma technology and two merited additional characterization. The mAb 1H10, an anti-head antibody, performed well in mouse passive transfer-viral challenge experiments, even if the administration of treatment was as late as 72 h post-infection, or the therapeutic dose was as low as 1 mg/kg. It has been well characterized that anti-head antibodies (Figure 8(A)) are in general specific to strains or even clade [20]. With the mutation of the HA gene of H7N9 viruses, the neutralizing antibodies generated by vaccination (A/Anhui/1/2013) do not react strongly with the newly emerging and mutated H7N9 viruses [49]. According to previous studies, three anti-head mAbs, L4A-14 [49], HNIgGA6 [20], and 1B2 [50], were developed against the HA of the H7N9 virus isolated in 2013. These mAbs demostrated neutralization potency against H7N9 virus isolated in 2016-2017. In our study, 1H10 showed a similar neutralization effect in vitro against the ZJU01 (LP H7N9 isolated in 2013) and the HP001 (HP H7N9 isolated in 2017), and exerted high protective efficacy when administrated prophylactically and therapeutically in the mouse model.

The mAb 2D1, a non-neutralizing mAb, provided protection by Fc-mediated antibody effector functions, such as ADCC (Figure 8(B)). Recent studies have demonstrated the importance of non-neutralizing antibodies in the clearance of influenza virus infections in vivo [51,52]. These non-neutralizing antibodies often showed extremely broad effects against almost all strains through ADCC, antibody-dependent cellular phagocytosis, antibody-dependent respiratory burst activity or complement-dependent cytotoxicity [46]. Although the antiviral activity of these anti-stem antibodies in vivo and in vitro is generally less potent than that of anti-head antibodies, they can exert better protective efficacy in vivo by cooperating with neutralizing antibodies.

Escape mutant variants were discovered and we found the epitope targets for mAbs 1H10 and 2D1 revealed that the MARM sites were located in antigenic site A. There were still differences between the two mAbs and viral escape mutations. The 1H10-recognized site (RRSGSS) is highly conserved and similar variants have been selected by the cross-reactive antibodies 5A6, 2C4, 4A2, 1A8 and 13-9-19-7 [46, 53, 54]. The conservation of antigenic site A explains the phenomenon that 1H10 targeting this site exerts cross-reactivity to ZJU01 and HP001. Additionally, the mAb 2D1 was sensitive to E124V and N249D. Among 1391 full-length HA proteins of H7N9 virus from GISAID, the residue substitutions (E124V and N249D) were not found in both human and avian isolates, which suggested that the mAb 2D1 were prospective against the circulating H7N9 viruses. However, the precise binding of our mAbs to H7-HA and the role of escape mutation sites should be studied further with such techniques as crystallography.

In conclusion, two murine antibodies, 1H10 and 2D1, exhibit significant prophylactic and therapeutic activities in mouse models of H7N9 infection with different mechanisms. The mAbs described here can be developed into potential antiviral therapeutics for H7N9 intervention.
